# Chronologically organized structure in autobiographical memory search

**DOI:** 10.3389/fpsyg.2015.00338

**Published:** 2015-03-25

**Authors:** Iva K. Brunec, Martin J. Chadwick, Amir-Homayoun Javadi, Ling Guo, Charlotte P. Malcolm, Hugo J. Spiers

**Affiliations:** ^1^Institute of Behavioural Neuroscience, Department of Experimental Psychology, Division of Psychology and Language Sciences, University College London, London, UK; ^2^Department of Psychology, University of Toronto, Toronto, ON, Canada; ^3^Department of Neuroscience, University of California, San Francisco, San Francisco, CA, USA

**Keywords:** episodic memory, temporal structure, contextual memory, event boundaries

## Abstract

Each of us has a rich set of autobiographical memories that provides us with a coherent story of our lives. These memories are known to be highly structured both thematically and temporally. However, it is not known how we naturally tend to explore the mental timeline of our memories. Here we developed a novel cued retrieval paradigm in order to investigate the temporal element of memory search. We found that, when asked to search for memories in the days immediately surrounding a salient cued event, participants displayed a marked set of temporal biases in their search patterns. Specifically, participants first tended to jump back in time and retrieve memories from the day prior to the cued event. Following this they then transitioned forward in time, and retrieved memories from the day after the cued event. This pattern of results replicated in a second experiment with a much larger group of participants, and a different method of cueing the memories. We argue that this set of temporal biases is consistent with memory search conforming to a temporally ordered narrative structure.

## Introduction

Humans may be unique in their ability to vividly and richly re-experience the important events from their past, an ability which has been referred to as “mental time travel” (Tulving, 2002). In order to allow travel through our own mental past, our autobiographical memories must have some kind of temporal structure or organizing principle, and indeed several studies have now demonstrated that we have access to such temporal information when retrieving memories (Burt et al., 2000, 2008; Janssen et al., 2006; Schulkind et al., 2012). What is less clear is how we tend to naturally explore the temporal dimension in memory. Most studies investigating memory exploration in autobiographical memory tend to find that people “move” from one memory to another based on shared thematic content rather than temporal proximity (Brown and Schopflocher, 1998; Burt et al., 2003; Lam and Buehler, 2009; Mace et al., 2013). However, given the clear temporal structure found among autobiographical memories, it should be possible to explore memories using that temporal information, rather than content-based information, effectively moving along our mental timeline. Here we aimed to investigate the nature of explicitly temporal exploration amongst autobiographical memories, with particular reference to the direction of exploration.

There are two competing hypotheses regarding possible biases in the direction of temporal memory exploration. The first hypothesis comes from studies investigating the retrieval of semantic information over a set of years. Participants were directed to either start at the beginning of the time period and work forward through memory, or start at the end and work backward. Increased accuracy was found when working backward through time (Whitten and Leonard, 1981; Loftus and Fathi, 1985). This suggests that participants may have a natural preference for exploring backward through their mental timeline. The second hypothesis comes from a more recent study by Skowronski et al. (2003), who investigated the ability of participants to judge the temporal order of pairs of autobiographical memories that had been recorded in a diary over a period of 9 weeks. They found that participants were more accurate at judging memory order when directed to search forward from one memory to the next, rather than backward in time. This latter result therefore suggests the opposite hypothesis that participants should display a bias for exploring forward through their mental timeline. This hypothesis assumes that people will have a natural inclination to construct a narrative that proceeds forward from a point after they have recalled the event, much like in the retrieval of a story narrative involving a succession of events (Mar, 2004).

Both of these conflicting sources of evidence come from studies that have specifically directed participants to explore either forward or backward through time, and therefore none of these studies directly speaks to the issue of how we naturally tend to explore our memories in time. The current study was designed to investigate any biases in the direction of memory exploration in conditions where participants were free to temporally explore in either direction. In order to accomplish this, we developed a novel autobiographical memory testing paradigm. Subjects were required to initially recall a salient personal event and then recall events that occurred in the days immediately around the cued event. Across two experiments, we used this paradigm to investigate possible biases in the temporal direction of memory exploration in order to compare the two competing bias hypotheses against a third null hypothesis of no temporal bias.

## Experiment 1

In order to determine whether there is a spontaneously elicited temporal direction of autobiographical memory recall, we designed a preliminary study in the form of structured interviews about participants’ autobiographical memories. Because of the great importance of autobiographical memory for the sense of self and the development and maintenance of close interpersonal relationships (Conway and Pleydell-Pearce, 2000; Bluck, 2003; Conway, 2005), we designed a test to probe participants’ memories for personal events.

### Methods

#### Materials and Stimuli

In a structured interview, participants were asked to recall seven personally experienced events. These included: when they last saw a friend from their hometown, a friend from primary school, a friend from high school, a friend from university, a co-worker from their first job after the age of 16, the friend they think they have known for the longest and a family member they only see at family gatherings or occasionally. For all cues, they were prompted to recall events that occurred longer than a week ago. The order of the cues was randomized across subjects.

#### Subjects

Twenty females participated. The subjects’ mean age was 26.0 (SD = 4.52) with the range between 19 and 37. All subjects were healthy native English speakers born in the UK. All gave informed written consent and were paid £7.50 per hour for their participation. Testing duration was approximately 20 min. The research was approved by the local research ethics committee.

#### Procedure

The test was carried out over the phone in the form of a structured interview and the phone calls were recorded and later transcribed. The time was scheduled ahead with the subjects who were asked to reserve 30–45 min in a peaceful environment.

The subjects were told that the recall task will involve thinking about their network of friends and acquaintances. They were then cued to recall the last occasion in which they had met one of the categories of friends/family listed above (Stimuli and Materials). We refer to this as the *Cued Event*. If the Cued Event had occurred in the last week, the subject was instructed to recall a different event involving the cued person that had occurred more than 1-week prior. Subjects were prompted to describe as many details as they could about that event. After each Cued Event recalled subjects were asked to recall events that occurred on the days *immediately* surrounding the Cued Event. This instruction therefore directs participants to temporally explore the memories around the Cued Event, with no constraint on the direction of exploration. Participants were free to search either forward or backward in time.

It was emphasized that they should report any specific elements that they clearly remember from the events in the surrounding days and not just assume what may have happened. Subjects were prompted to recall everything they could remember about the surrounding days until they indicated that they could not recall any further events.

The procedure was initially explained to subjects with an example, asking them to recall when they last saw their nearest neighbor, to ensure they understood the task instructions.

#### Analysis

To test whether there was a bias in the temporal direction of memory search from the Cued Event, we calculated a bias score. Participants were instructed to recall as many events as they could from the days immediately surrounding the Cued Event, meaning that the number of events recalled varied across trials. The bias score was calculated separately for the first event recalled (*Event 1*) and for any second event recalled (*Event 2*). Subjects rarely described a third event (12 subjects out of 20 recalled a third event on an average of 19.3% of trials), thus these were not considered in the analysis. The bias score was the number of trials for which an event was recalled from the day *after* the Cued Event minus the number of trials for which subjects recalled events from the day *before* the Cued Event, divided by the total number of trials for which events were recalled. For example, if Event 1 occurred on the day before the Cued Event for 5 out of 7 of the cues and on the day after for the remaining 2 out of 7, the bias score for Event 1 for the subject would equal –0.429 [as calculated from (2–5)/7]. This provided a normalized measure of bias where –1 indicates that all trials were recalled from the day before the Cued Event (i.e., searching memories backward in time), and +1 indicates that all trials were recalled from the day after the Cued Event (i.e., searching memories forward in time). Statistical analyses of the data were carried out with SPSS.

**FIGURE 1:**
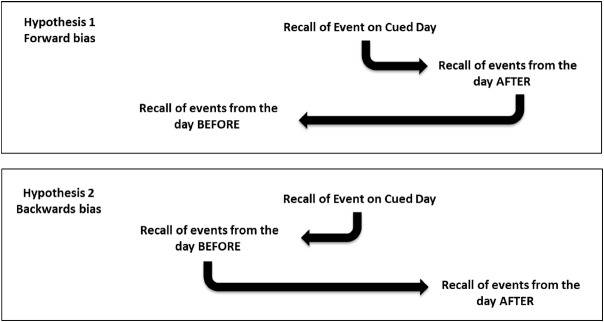
**Two hypotheses regarding the nature of autobiographical memory recall.** Arrows indicate the transition from retrieval of events on one day to retrieval of events on another day. The diagrams show the possible patterns when events on both the day before and after are recalled. Where subjects only recall one of the days, the null hypothesis predicts an equal distribution of recalls of the events on the day before and after, Hypothesis 1 a bias to the day after and Hypothesis 2 a bias to the day before.

We tested three alternative hypotheses about the data by examining the bias scores (see Figure 1). If the bias scores were no different to 0, this would mean the null hypothesis (no bias) would not be rejected. If the bias scores for Event 1 were significantly greater than 0 this would support the Hypothesis 1 that subjects follow the causal chain of events to activate memories for events in the future, and subsequently return to the day before. However, if the bias scores for Event 1 were significantly less than 0 this would support the second hypothesis that subjects begin by searching their stored representations of the events occurring before the Cued Event and then proceed forward in time to the day after the Cued Event. We further hypothesized that Event 2 would show a complementary bias in the direction of search as outlined in Figure 1.

### Results

**FIGURE 2:**
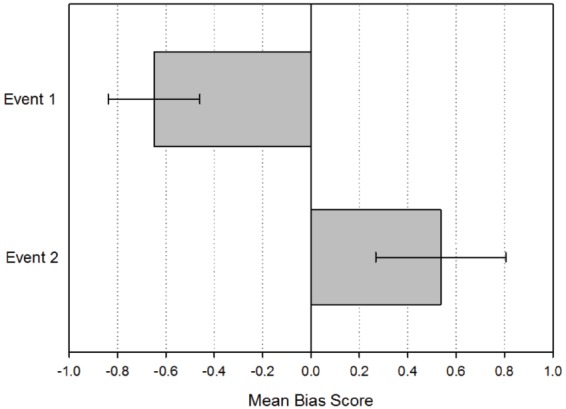
**Mean bias scores for the first event recalled (Event 1) and the second event recalled (Event 2).** Negative mean bias refers to past and positive mean bias refers to future. Error bars indicate 95% confidence interval.

Subjects were able to describe details from at least one event that occurred in the days immediately around the Cued Event (Event 1) on 70.7% of trials (SD = 22.35), and just over half recalled a second event (Event 2: mean percentage of trials for which an event was recalled = 54.5%, SD = 27.51). The mean bias score for Event 1 was –0.649 (SD = 0.40) and the mean bias score for Event 2 was 0.537 (SD = 0.57), see Figure 2. One-sample *t*-tests confirmed that the bias score for Event 1 was significantly less than 0 [*t*(19) = –7.173, *p* < 0.001, 95% CI (–0.838, –0.460)] and the bias score for Event 2 was significantly greater than 0 [*t*(19) = 4.184, *p* = 0.001, 95% CI (0.268, 0.805)]. The difference between bias scores was also significant [*t*(19) = –6.761, *p* < 0.001, 95% CI (–1.553, –0.819)]. Thus subjects showed a bias toward initially recalling an event in the day before the Cued Event and subsequently, if they then recalled a second event, they showed a bias to recalling an event occurring the day after the Cued Event. Only one subject showed an overall forward bias for Event 1 (bias score = 0.33) and two subjects showed no bias (bias score = 0.0). This pattern was reversed for Event 2, where two subjects showed an overall backward bias (–0.2 and –1) and four subjects showed no bias (bias score = 0.0).

Such temporal structure was apparent in participants’ responses:

“The last time I saw [friend from university] would be just before Christmas, I threw a Christmas party. /…/ I remember being super stressed before the party because we had this statistics exam and I’d just done it so I remember feeling very relieved and happy on the day of the party. /…/ And then I remember my sister came home the day after the party and I cooked a meal for my family.”

and

“I last saw [the friend known for the longest] on a Friday in early September 2009. It was another friend’s wedding. I remember having the day off work, that’s how I know it was a Friday. /…/ The day before the wedding, I remember I had my fifth date with my boyfriend who was helping me choose between a purple and a black dress to wear to the wedding. And then the day after the wedding, on the Saturday, I remember going for a long walk. I remember taking the train from [the wedding venue], and then a taxi to get to the countryside. So then I took a long walk in the brush in the farmland.”

Our results therefore show a bias to initially recalling the day before when exploring autobiographical memories around a salient event. However, it is possible that there is a bias in the amount of information recalled in the day before compared to the day after, which could explain our results without there being any bias in the natural direction of memory exploration. To test this we examined the mean number of verifiable details recalled for each of the days around the Cued Event (for example, the location, the time, and the people present). There was no difference between the number of details retrieved for the days around the salient event [day before = 1.98 details (SD = 1.14), day after = 1.57 details (SD = 1.47), *t*(19) = 1.385, *p* = 0.182, 95% CI (–0.211, 1.034)]. Thus, while subjects were more likely to return to the day before when searching their autobiographical memory, they did not recall more from that day than the day after, if they did also recall the day after. We did find a significant increase in the number of details recalled from the Cued Event compared to the days surrounding this event [Cued Event = 5.32 details (SD = 1.58), *t*(19) = 11.43, *p* < 0.001, 95% CI (2.894, 4.192)]. However, given that this event was retrieved with the aid of a salient social cue, this result is unsurprising.

### Discussion

In summary, we find that when people search their memory for events on the days around a cued event they show a bias in the direction of memory search, exploring backward in time to the day before. However, following this initial backward search, they then show a bias in progressing forward, to the day following the cued event. To our knowledge this bias in autobiographical memory search has not been previously reported and is consistent with hypothesis 2 in our formulation of possible outcomes (Figure 1). The absence of any differences in the number of event details recalled on the days before or after further indicates that this bias affect is not due to an ease at recalling more information, but points rather to a genuine temporal bias in the direction of memory search.

Having established evidence for an effect, we next sought to: (a) replicate our findings in a larger population, (b) test male subjects, and (c) assess the effects without the interaction with an interviewer. While our interviews were carefully constructed to avoid cueing the subject to recall events from the past or future days we cannot rule out the possibility that subtle social cues may have influenced the subject’s behavior.

## Experiment 2

Based on the promising results of the first experiment, we adapted the task as a controlled and structured investigation into the organization of autobiographical recall. Instead of describing events in long-form sentences, the participants were only required to think of the first, then the second, and then the third word that came to their minds when thinking about the days surrounding an event.

### Methods

#### Participants

We were able to collect data from 60 male and 60 female participants. A *post hoc* power analysis (using GPower 3.1; Faul et al., 2007) for the two groups *t*-test revealed that, for these sample sizes and a two-tailed alpha of 0.05, medium gender effects (*d* = 0.5) are detected with a power of 0.78. This is close to the power level of 0.80 that is generally deemed adequate in psychological research (see Cohen, 1992). The participants’ mean age was 22.5 years (range 18–35 years). All participants were native or highly fluent English speakers and gave their informed consent before participating in the study. All participants were tested according to the local ethics committee guidelines. They received course credit or £3 for their time (approximately 15–20 min).

#### Procedure

MATLAB (2011b, MathWorks) and Psychtoolbox (v3, Brainard, 1997) were used to collect demographics, display the memory cues, and store and re-display the words provided by the participants to describe their memories.

The participants’ demographics were first collected. Afterward, instructions were displayed to them, explaining to them that they will be asked to think about the events they experience with people from their life. They were told that they would be given some time to think about each person and then asked to write down the first word that came to mind. They were then told that they would be asked to think about the days *immediately around* the event they had just recalled: “*You will then be asked to think about any other experiences you recall from the days immediately around the event you just thought about. You will be asked to write down the first, then the second, then the third word that comes to your mind when thinking about the days around the event*.”

It was emphasized to participants that they should think about the days immediately surrounding the event they had just recalled—critically, however, the words “before” and “after” were never used to avoid biasing them. They were prompted to try their best to recall an event, and spend some time thinking about it even if they could not immediately recall anything. They were instructed to think of a different person if an event had already been brought to mind in a previous trial or if it happened within the past week.

Each memory cue was displayed for 15 s which were counting down on the screen, again preceded by a practice trial. During this time, the participants were asked to recall the displayed event and think about it. Below the event, the following prompt was always displayed: “*If you have experienced this event in the past week or you have described it in a previous trial, please try to think of a different time that this event happened*.”

They were then asked to type the first word that came to their minds from the day of that event: “*Please write down the first word that comes to mind when thinking about this event and press enter*.”

After this, they were asked to recall the first, then the second, then the third word that came to their mind from the days surrounding this event:

“*Now think of anything that you can remember that happened in the days immediately around the event you just thought about. Please write down the first word that comes to mind when thinking about the DAYS AROUND this event and press enter*.”

After they entered the word, they were taken to the next screen, where the same prompts were displayed for the second and third words. This was repeated for each of the nine memory cues. After all trials were completed, they were then told that they will be shown each of the three words they typed, one at a time, and they will have to decide whether each of them is related to the day before or to the day after each event. They were instructed to press the left arrow key if the word described an event on the day prior to the anchor event and the right arrow key if it described an event that occurred after the anchor event. They were instructed to press the “up” arrow key if they typed “none.” They were then told that they will have to provide some context for everything they typed after they finish all trials.

After the participants had indicated the temporal direction for all words, they were finally shown a screen containing all four words that they had entered for the event itself and each of the surrounding events and asked to provide a sentence to describe the context surrounding these words. After they had described each of the events, the experiment was completed.

### Results

The three words provided by the participants for the days surrounding each event were coded according to whether they were referring to days before or after the event. Similarly to Experiment 1, if the participant indicated that a word was referring to the days before an event, this was coded with –1 and if it was referring to the days afterward, it was coded with 1. The average of all provided responses was calculated for each of the events, relying on the context provided by the participants. The three words provided by participants did not necessarily refer to three distinct events. As we aimed to examine recall direction for specific events, any words that referred to the same event were collapsed into a single event score. The same analysis as for Experiment 1 then applied.

We were initially only interested in the first event reported by the participants, which was also expressed in the instructions, where an example of words provided was only given for an event on “one of the days surrounding the event.” However, participants commonly provided more than one event. Event 1 was provided in 96.4% of trials and Event 2 in 47.7% of all trials. six females and one male could only recall one event from the days surrounding the Cued Event. Only 8% of all trials included a word referring to a third event occurring in the days surrounding the memory cue. These were not included in the analysis as the statistical power of the test would be insufficient.

The average bias for Event 1 was –0.13 (SD = 0.50) and the average bias for Event 2 was 0.31 (SD = 0.56). The average bias score for Event 1 was –0.164 (SD = 0.51) for females and –0.093 for males (SD = 0.50). For Event 2, the average bias score for females was 0.37 (SD = 0.57) and 0.24 for males (SD = 0.55). An independent samples *t*-test showed that there were no significant gender differences in the bias on Event 1 [*t*(118) = –0.775, *p* = 0.440, 95% CI (–0.255, 0.111)] or Event 2 [*t*(104) = 1.248, *p* = 0.215, 95% CI (–0.080, 0.352)]. Females provided 97.0% of responses on Event 1 (SD = 7.61) and males provided 95.7% (SD = 10.25). For Event 2, females provided 46.9% (SD = 29.0) of responses and males provided 48.4% (SD = 29.5). There was no gender difference in the proportion of provided responses on Event 1 [*t*(118) = 0.768, *p* = 0.443, 95% CI (–0.020, 0.046)] or Event 2 [*t*(118) = –0.287, *p* = 0.774, 95% CI (–0.121, 0.090)]. All subsequent analyses were collapsed across males and females on the basis of this non-significant outcome.

**FIGURE 3:**
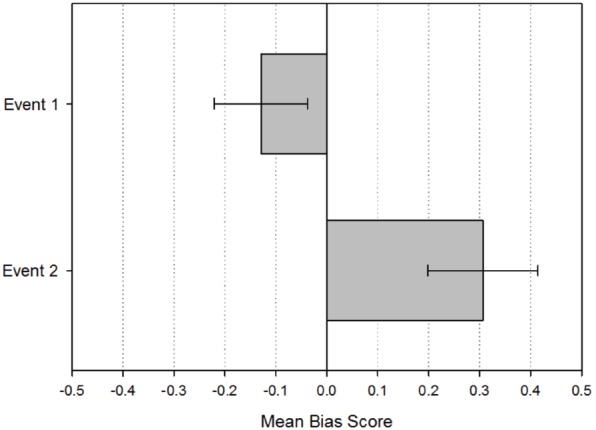
**Mean bias scores for the first and second events recalled by the participants.** Error bars indicate 95% confidence interval.

Figure 3 depicts the bias scores for each event.

Two one sample *t*-tests were carried out to establish whether the bias scores for Event 1 and Event 2 significantly differed from 0. Bias scores for Event 1 were found to be significantly lower than 0: *t*(119) = –2.785, *p* = 0.006, 95% CI (–0.221, –0.037), while they were significantly greater than 0 for Event 2: *t*(105) = 5.617, *p* < 0.001, 95% CI (0.198, 0.415). The difference between the bias scores for the two events was also significant as indicated by a paired-sample *t*-test: *t*(105) = –4.931, *p* < 0.001, 95% CI (–0.615, –0.262). The data are therefore consistent with the results of Experiment 1, suggesting that participants initially recalled the days before a Cued Event and then searched forward in time.

While the initial backward search bias is a clear temporal bias, this might not be the case for the subsequent forward bias for Event 2. It is possible that this bias might be due to limitations in the total number of memories available per day, rather than any genuine temporal biases. For example, if we assume that participants have access to a maximum of one event per day, then the constraints of the task will automatically produce this apparent temporal bias. This is because they are limited to searching memory in the two days immediately surrounding the cued event. As soon as they have retrieved a memory from the day before the cued event, they do not have access to any more events on that day. The only possible remaining memories will be on the day after the cued event, forcing participants to move forward in time to continue searching for a second memory. This pattern of results would appear to follow a temporal search from past to future, but this would merely be an artifact of the underlying memory constraints, and not a true temporal bias. We therefore refer to this alternative account as the “limited memories” hypothesis. We next explored the precise pattern of memory transitions in order to determine whether the apparent bias for Event 2 is genuinely temporal in nature.

To accomplish this, we made use of the fact that there were many trials where Event 1 was recalled from the day after the cued event. This property is important, because the limited memories account would predict a symmetrical pattern of memory transitions. In other words, if the only factor driving the apparent transition from past to future is a limit in the number of accessible memories, then we ought to see an equally strong effect in the opposite direction, from future to past, on those trials where Event 1 was retrieved from the day after the cued event. In contrast, if there is a genuinely temporal explanation for the bias, then there should be a significantly greater probability of transitioning from the past to the future, then from the future to the past.

To test these two hypotheses, the probability of forward and backward transitions was calculated. A *forward transition* is defined as a trial where the first recalled event occurred before the Cued Event, and then the second recalled event transitions forward in time to the day after the Cued Event. Similarly, a *backward transition* is where the first recalled event occurred after the anchor event, and then the second transitions backward in time to the day before the anchor.

Forward transition probability was calculated as the number of forward transition trials divided by the total number of trials where the first recalled event was before the anchor, and a second event was provided. Backward transition probability was calculated as the number of backward transitions divided by the total number of trials where the first recalled event was after the Cued Event, and a second event was provided. For example, if a participant showed a forward transition (“before” to “after”) on four trials, and showed no transition (“before” and “before”) on three trials, their forward transition probability would be 4/(4 + 3) = 0.57. This would indicate that if a participant initially recalls an event before the Cued Event.=, they have a likelihood of 57% of transitioning through time to events after the Cued Event. If the overall trend is indeed that participants prefer to move forward in time, the *forward* transition probability for the events provided should be significantly greater than *backward* transition probability. The forward and backward transition probabilities were calculated for every participant, and these two sets of transition probabilities were compared with a paired samples *t*-test.

The mean forward transition probability was 0.629 (SD = 0.311) and the mean proportion of backward transitions was 0.425 (SD = 0.351). A paired samples *t-*test confirmed that there were significantly more forward transitions than backward ones: *t*(105) = 5.137, *p* < 0.001, 95% CI (0.125, 0.282). An independent samples *t*-test suggested that there were no significant differences between males and females in either forward [*t*(104) = –0.289, *p* = 0.773, 95% CI (–0.138, 0.103)] or backward transition probabilities [*t*(104) = –0.588, *p* = 0.557, 95% CI (–0.176, 0.095)].

We next considered the possibility that affective valence of a recalled event might have influenced the direction of recall (Berntsen and Rubin, 2002; Berntsen et al., 2011; Szpunar et al., 2012). The context of each retrieved event was analyzed and coded with 0 if the event was neutral, 1 if it was positive, and –1 if it was negative. Two independent raters individually coded the valence and agreed on a value by discussing points of disagreement. In order to establish whether the valence (positive, negative, or neutral) was predictive of the participants’ key presses (–1 for the day before and 1 for the day after) for the first event, we carried out a repeated measures ANOVA where we compared the bias scores for each of the three valence categories. There were no significant differences in the bias scores depending on the emotional content of the first recalled event: *F*(2,128) = 1.726, *p* = 0.182.

Finally, we compared the main results from experiments 1 and 2 in order to determine whether there were any significant differences in effect size between the two. An independent samples *t*-tests revealed that the negative bias on Event 1 found in interviews was significantly greater: *t*(138) = 4.358, *p* < 0.001, 95% CI (0.284, 0.756). In contrast, there was no significant difference in the bias found for Event 2 between Experiments 1 and 2: *t*(124) = –1.675, *p* = 0.096, 95% CI (–0.502, 0.042). The proportion of “None” responses was significantly greater in Experiment 2 relative to Experiment 1: *t*(138) = –8.91, *p* < 0.001, 95% CI (–0.31, –0.20). There was no significant difference in the proportion of “None” responses for Event 2: *t*(138) = –1.29, *p* = 0.199, 95% CI (–0.23, 0.05). As only women were tested in the interview setting, a further *t*-test was done comparing interview bias scores with just female bias scores in Experiment 2, again showing that the negative bias on Event 1 was significantly greater in the interviews: *t*(78) = 4.30, *p* < 0.001, 95% CI (0.281, 0.768), but there was no significant difference on Event 2: *t*(71) = –1.085, *p* = 0.282, 95% CI (–0.461, 0.136).

### Discussion

These results replicate those of Experiment 1, and demonstrate a temporal bias in memory exploration, such that participants tend to initially search back in time until they find one memory, then proceed forward in time until they find another memory. They further show that the forward transition between memories is a genuinely temporal bias, rather than being due to other explanations such as memory limitations. Finally, these results demonstrate that the temporal bias effects hold across two very different means of cueing the memories, and are not simply an artifact of interview-based memory cueing.

## General Discussion

The two experiments reported here provide, to the best of our knowledge, the first systematic investigation of the order of recall of events in the days immediately surrounding a single cued event. The results suggest that there is a significant tendency to chronologically structure autobiographical recall such that we prefer to first recall events before a salient memory anchor and then mentally travel forward in time and continue to recall events after it. This initial backward bias, followed by a forward transition tendency, was observed regardless of the number of details participants provided for each of the surrounding days (Experiment 1) or the valence of the details (Experiment 2). Thus the effect may be assumed to exist separately from the vividness or richness of details and the emotional context of the memory.

We propose that this pattern of results is consistent with the idea that autobiographical memories are linked together into a coherent narrative structure (Conway and Pleydell-Pearce, 2000; Singer and Blagov, 2004; Conway, 2005; McAdams, 2006). When subjects are required to retrieve memories from the days around a central cued event, they naturally go back to the beginning of this “narrative window,” and then systematically work forward in time from this start point. Such a mechanism would explain both facets of our results—both the initial jump back in time, and the subsequent transition forward in time.

This pattern of results is consistent with that reported by Loftus and Fathi (1985) and Whitten and Leonard (1981), who showed that backward search occurs spontaneously and is more successful than forward search. However, the present finding can also reconcile the discrepancy between these studies on the one hand and the results obtained by Skowronski et al. (2003) on the other hand, who report a forward bias—after searching backward to retrieve the sequence of events surrounding the anchor, their narrative proceeded in the forward direction. It might also explain why the initial backward bias was significantly more pronounced in Experiment 1 than Experiment 2. The conversational testing format used in the inverview-based procedure of Experiment 1 may have enhanced the natural tendency to produce a structured narrative (Adler and McAdams, 2007), thereby leading to a stronger temporal bias.

One possible limitation of the current study was that the retrieved memories were not externally validated as real memories. While spontaneous confabulation is rare in non-clinical populations and tends to be restricted to patients with frontal lobe damage (Fischer et al., 1995; Moscovitch and Melo, 1997), false or distorted memories appear to be common and difficult to identify (Bahrick et al., 1996; Loftus, 2000). We cannot therefore prove that the retrieved memories were always accurate. Importantly however, our main finding of temporal biases in the direction of memory search still holds, regardless of how accurate those memories actually are. A further limitation is that the time-scale of memory retrieval was restricted to memories in the days immediately surrounding a cued event. We therefore cannot be sure that the same temporal biases would also hold for other time-scales, over weeks, months or years (or conversely, over events within a single day). Further study will be required to determine whether this is a general effect regardless of the temporal distance between the memories.

One important question raised by these results, and others in the autobiographical memory literature, is how we encode the temporal information of new memories. Given that there is a clear temporal structure among our autobiographical memories, and a clear bias in the way that we explore these memories, there must be some mechanism by which this temporal information is stored. Research on temporal coding of sequence recall has produced compelling evidence for models such as the Temporal Context Model (Howard and Kahana, 1999, 2002; Polyn and Kahana, 2008; Hsieh et al., 2014), which suggest that the order of events can be coded by a slowly shifting neural representation of temporal context.

According to the Temporal Context Model, the retrieval of one item from a series facilitates the recall of the subsequent item. Thus, one interpretation of this model is that it would predict an initial recall of the day after the cued event. This is not what we observed. However, the focus of the Temporal Context Model is temporal coding over short periods of time, and it has not yet been adapted to explain the existence of temporal structure over much longer periods of time. We propose that the cued event acts as an anchor which activates the representations of events on the surrounding days. The cue “days immediately surrounding the cued event” leads subjects to set their retrieval orientation to the start of the experiences to be recalled. Once the first day is recalled, recall spreads forward to the day after. This forward driven recall may occur via mechanisms described in the Temporal Context Model (Howard and Kahana, 1999, 2002; Polyn and Kahana, 2008). Determining whether similar mechanisms might explain temporal coding over longer time periods will be an important direction for future work. Conversely, the recall of elements within the same event boundaries such as events within the same day, should be further explored.

In summary, we provide the first evidence of a bias in the temporal direction of autobiographical memory exploration, such that we preferentially recall the days before a cued event and then proceed by recalling the days after the initial autobiographical anchor. This temporal bias is consistent with a narrative structuring of memories, even under circumstances that minimized any social benefits of narrative structuring. We suggest that these results are therefore consistent with an automatic structuring of memories into a coherent narrative, consistent with current theories of autobiographical memory (Conway and Pleydell-Pearce, 2000; Conway, 2005).

### Conflict of Interest Statement

The authors declare that the research was conducted in the absence of any commercial or financial relationships that could be construed as a potential conflict of interest.
